# Cremation

**Published:** 1881-03

**Authors:** 


Cremation.—Whether cremation is considered from an economical or hygienical standpoint, it is well worthy of calm and dispassionate investigation, and we are glad to see some of our contemporaries discussing it with intelligence and fairness. As all who are born must die, it is certain that some disposition will be made of their mortal remains, and the question naturally suggests itsself to those living in large cities, particularly, what is the *best* method or mode of disposing of their dead?

In rural districts, the old method of burial will probably never be abandoned entirely for any other; certainly not unless their populations should be vastly increased beyond what may be reasonably expected at the present time; and as no hygienic requirements for other disposal of their dead, than by burial, are likely to arise, in the usual order of things, nothing further need be added on that point. But the question of disposal of the dead in large and densely populated cities, is one of great and growing importance in many of those communities.

As intra-municipal interments have been pretty generally abandoned, unquestionably in the interest of hygiene, in most of our cities, and large towns, it will only be necessary to speak of the matter in general terms, in this brief editorial. Should crematories be erected either by corporate or individual enterprise, they would prove good investments, were the burial of the dead abolished, as even the poorest in a community would find it vastly cheaper to cremate than bury their dead! And a general adoption of that method of disposing of the dead of cities and large towns, would save millions of money annually to their inhabitants. That a vast improvement would result in the sanitary condition of all such communities, from the adoption of cremation cannot admit of reasonable question; which to some of them, as New Orleans, for example, where, owing to peculiarity of topography and locality, graves cannot be made of sufficient depth below the surface, cremation would seem to be loudly called for, both on account of health and economy of means! We hope to find time to revert to this subject again.

